# Exploring Biochemical Predictors of Reversible Mild Cognitive Impairment: A Longitudinal Approach

**DOI:** 10.1002/alz70860_106396

**Published:** 2025-12-23

**Authors:** Monisha S, Thomas Gregor Issac

**Affiliations:** ^1^ Centre for Brain Research, Indian Institute of Science, Bengaluru, Karnataka, India; ^2^ Centre for Brain Research, Indian Institute of Science, Bangalore, Karnataka, India

## Abstract

**Background:**

MCI is considered to be the intermediate stage between normal aging and early dementia. Many studies have focused on finding predictors for the progression of MCI. Very few studies have focused on reversible MCI. The biochemical markers are those related to neuroinflammation and neurodegeneration which could potentially serve as a predictor of reversion from cognitive impairment. The study aims to examine the biochemical factors and their association with reversible MCI.

**Method:**

The study included 48 participants from the Tata Longitudinal Study of Ageing (TLSA), an ongoing cohort study of individuals aged ≥ 45 years. Data from baseline and a follow‐up visit after 18 months were utilized in this analysis. The detailed biochemical assessments were done using blood samples collected from the participants. The Clinical Dementia Rating (CDR) Scale was used to identify the participants with reversible MCI. The paired t‐test/ Wilcoxon signed‐rank test was used to find differences between two related time points.

**Result:**

The prevalence of reversible MCI in the population was found to be 5.1%. The Wilcoxon signed‐rank test revealed a significant change in homocysteine levels (Z=‐3.289; *p* = 0.001), vitamin D (Z = ‐1.999; *p* = 0.046), serum triglycerides (Z=‐2.169; *p* = 0.030)), and serum Very Low‐Density Lipoprotein (VLDL) (Z = ‐2.618; *p* = 0.009) between baseline and follow‐up. The results suggest that biochemical markers improved significantly over 18 months in participants with reversion from MCI to normal cognition.

**Conclusion:**

These results highlight the importance of metabolic and nutritional factors in promoting cognitive health and modifying these could be a key strategy in the management of MCI. Given the prevalence of reversible MCI in the study population, further research is required to understand the underlying mechanisms and the potential for therapeutic interventions targeting these biochemical factors to support cognitive recovery in individuals with MCI.

